# The Effect of Pulse Frequency on the Microstructure and Corrosion Resistance of an AZ31B Magnesium Alloy Composite Coating with Electron-Beam Remelting and Micro-Arc Oxidation

**DOI:** 10.3390/ma18091962

**Published:** 2025-04-25

**Authors:** Yinghe Ma, Zhen Yu, Jinpeng Zhang, Yonghui Hu, Mengliang Zhou, Jinhui Mei, Zhihui Cai, Wenjian Zheng, Jianguo Yang

**Affiliations:** 1 Institute of Process Equipment and Control Engineering, College of Mechanical Engineering, Zhejiang University of Technology, Hangzhou 310023, China; mayh@zjut.edu.cn (Y.M.); 18005730662@163.com (Z.Y.); zhjp2323@163.com (J.Z.); huyh20010717@163.com (Y.H.); zml1956562840@163.com (M.Z.); 15267672316@163.com (J.M.); zwj0322@zjut.edu.cn (W.Z.); 2 Engineering Research Center of Process Equipment and Remanufacturing, Ministry of Education, Zhejiang University of Technology, Hangzhou 310023, China; 3 Wenzhou Special Equipment Inspection & Science Research Institute, Wenzhou 325800, China

**Keywords:** AZ31B magnesium alloy, micro-arc oxidation, electron-beam remelting, corrosion resistance, pulse frequency

## Abstract

This study presents a systematic investigation into the influence of pulse frequency on the micro-arc oxidation (MAO) coating of AZ31B magnesium alloy following electron-beam remelting (EBR). The morphology, thickness, and corrosion resistance of the EBR-MAO composite coating were meticulously analyzed across various pulse frequencies (100 Hz, 200 Hz, 300 Hz, 400 Hz) employing scanning electron microscopy (SEM), X-ray diffraction (XRD), and electrochemical measurement techniques. The results show that as the pulse frequency escalates from 100 Hz to 400 Hz, the average thickness of the EBR-MAO composite coating diminishes from 41.1 μm to 38.5 μm, reduced by 6.7% compared to 10.4% in the MAO coating. Concurrently, the porosity exhibits a reduction from 1.93% to 1.35%, accompanied by a densification of the coating’s structure. High pulse frequencies yield coatings with enhanced smoothness and fewer defects. Notably, the corrosion resistance of the coatings demonstrates significant improvement at higher frequencies (400 Hz) compared to their lower-frequency (100 Hz) counterparts, as evidenced by a tenfold increase in corrosion current density. This research underscores the pivotal role of pulse frequency in optimizing the protective qualities of MAO coatings on magnesium alloys.

## 1. Introduction

Magnesium alloys, as an important class of lightweight structural materials, possess advantages such as weight ratio, good ductility, excellent processability, and low density [1,2,3]. Additionally, the degradation of magnesium alloys in the human body releases magnesium ions with good biocompatibility, making them highly promising in various fields including engineering machinery, aerospace, biomedical applications, the automotive industry, sports equipment, and electronic devices. As such, magnesium alloys are regarded as one of the most promising metal materials of the 21st century [4,5,6].

However, due to their chemically active nature and the presence of secondary phases such as aluminum and manganese, magnesium alloys have a stable potential of −1.5 to −1.6 V in seawater, making them highly susceptible to surface oxidation and galvanic corrosion [7,8,9]. This results in poor corrosion resistance in various media, greatly limiting their practical applications in engineering. Therefore, to enhance corrosion resistance and extend service life, surface treatment is essential. Commonly used surface-treatment methods include electrochemical treatments, electroplating and electroless plating, organic coating treatments, and surface-modification technologies [10,11,12,13].

In terms of improving surface properties, W. Li et al. [14] significantly enhanced the wear and corrosion resistance of AZ31B magnesium alloy micro-arc oxidation (MAO) coatings through laser surface melting (LSM) pretreatment. After LSM treatment, the grain size of the substrate was significantly refined, the average pore area of the MAO coating decreased to 0.84 μm^2^, porosity was reduced to 7.14%, the wear rate decreased by 50%, the self-corrosion current density was reduced by two orders of magnitude, and polarization resistance increased by one order of magnitude. Y.J. Wang [15] further confirmed that the grain size and uniformity of magnesium alloys are key factors affecting their corrosion resistance. S.Y. Huang et al. [16] found that introducing an anodic pause time (as opposed to a cathodic pause) in the MAO process significantly improved the corrosion resistance of the coating. H.R. Dong et al. [17] systematically studied the coating-formation ability and performance of MAO coatings in different types of single electrolytes, offering deeper insight into the coating-formation mechanism. L.J. Bai et al. [18] employed an asymmetric bipolar pulse MAO technique to fabricate black, corrosion-resistant, and light-absorbing ceramic coatings on AZ31 magnesium alloy and investigated the influence of positive pulse voltage on coating color and corrosion resistance. D.Y. Wang et al. [19] aimed to regulate the degradation rate of AZ31B magnesium alloy for use in biomedical implants by creating microporous structures with varying pore diameters and spacings on the MAO coating surface using femtosecond lasers. H.K. Pan et al. [20] first applied MAO to AZ31 alloy and then conducted cathodic deposition (CD) in an ethylene glycol organic electrolyte, successfully preparing composite coatings with excellent corrosion and friction resistance. S. Sun et al. [21] sequentially applied laser remelting, MAO treatment, and layered double hydroxide (LDH) sealing treatment on AZ31 alloy, significantly enhancing the corrosion resistance of the composite coatings.

Although LSM is widely applied, it poses challenges when used on magnesium alloys due to their sensitivity to oxidation during laser processing, which may negatively affect subsequent MAO treatment. In contrast, electron-beam remelting (EBR) technology has recently attracted increasing attention. EBR has gained wide interest due to its unique advantages. Compared with other surface-modification techniques, EBR offers several notable advantages [22,23]: (1) high energy density and rapid heating with a small heat-affected zone, beneficial for preserving the substrate properties; (2) precise control over processing parameters; (3) rapid solidification conducive to grain refinement and property enhancement; and (4) operation in a vacuum environment, which avoids high-temperature oxidation and facilitates the formation of dense, high-quality modified layers. G.H. Zhao et al. [24] used EBR to improve the surface performance of stainless steel, achieving enhanced surface morphology, reduced roughness, refined grains, and increased dislocations density. J.Y. Yao et al. [25] applied EBR to improve the surface properties of Inconel 625 nickel-based alloy, and the modified material showed significant reductions in oxidative and adhesive wear compared to the untreated material.

At present, single surface-treatment methods can no longer meet the complex and demanding requirements of practical engineering applications. The fabrication of high-performance composite coatings by combining multiple surface-treatment techniques has gradually become a research hotspot and development trend in the field of surface engineering. Previous studies have shown that EBR significantly improves the density and corrosion resistance of MAO coatings on AZ31B magnesium alloy [26]. Given that electrical parameters in the MAO process—especially pulse frequency—have a major influence on the microstructure and properties of the coatings, further in-depth research is necessary to optimize these parameters and enhance the overall performance of the composite coatings.

In this study, AZ31B magnesium alloy samples treated with EBR were subjected to MAO treatment under different pulse-frequency conditions. The effects of pulse frequency on the microstructure and composition of the coatings were systematically analyzed, and the corrosion resistance of the resulting composite coatings was thoroughly evaluated. Based on the experimental results, the influence mechanism of pulse frequency on the performance of EBR-MAO composite coatings was elucidated, providing valuable theoretical guidance and technical insights for optimizing coating processes and improving the comprehensive performance of magnesium alloy materials.

## 2. Experimental Details

### 2.1. Preparation of EBR AZ31B Magnesium Alloy Samples

AZ31B magnesium alloy plates with a thickness of 2 mm were machined into specimens measuring 33 × 33 mm. The surfaces were sequentially ground using 320#, 600#, 800#, and 1000# SiC abrasive papers until smooth. The specimens were then ultrasonically cleaned in acetone and ethanol for 3 min each to thoroughly remove surface oils and other contaminants. Electron-beam remelting (EBR) was performed on the prepared AZ31B specimens using a THDW-4 electron-beam welding machine manufactured by Guilin Sida Technology Co., Ltd. (Guilin, China). The EBR process parameters were as follows: an accelerating voltage of 65 kV, a welding speed of 200 mm/min, a beam overlap rate of 50%, and an electron-beam current of 0.5 mA.

### 2.2. Preparation of EBR-MAO Composite Coatings

**Figure 1:**
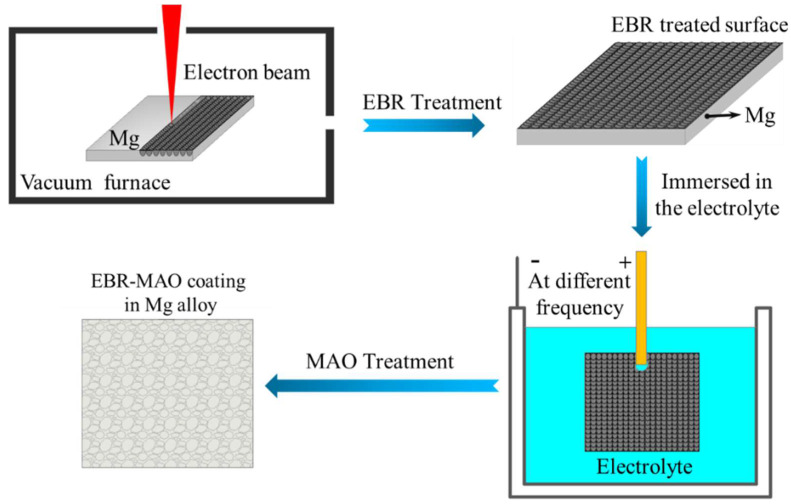
Flow chart of EBR-MAO process.

After the EBR process, the samples were further ground and polished using sandpapers and a 0.25 μm diamond suspension polishing agent to ensure a smooth surface. The samples were then ultrasonically cleaned in alcohol and acetone, followed by drying for subsequent use. The micro-arc oxidation (MAO) treatment was performed in a silicate-based electrolyte system (Na_2_-SiO_3_: 8 g/L, NaF: 0.5 g/L, KOH: 1 g/L, pH 12–13) with an electrolyte volume of 6 L. The MAO process was conducted under constant-voltage mode with the following parameters: a voltage of 520 V, a duty cycle of 20%, a treatment time of 20 min, and pulse frequencies of 100, 200, 300, and 400 Hz. The MAO sample numbers at different frequencies are as follows: M100, M200, M300, M400. The EBR-MAO sample numbers at different frequencies are as follows: EM100, EM200, EM300, EM400. During the MAO process, the electrolytic cell was cooled using circulating water to maintain the temperature below 40 °C. After the MAO treatment, the samples were rinsed with deionized water for 3 min to remove residual electrolytes. A schematic diagram illustrating the preparation process of the EBR-MAO composite coatings is presented in Figure 1.

### 2.3. Characterization

The surface roughness of the substrate with and without electron-beam remelting (EBR) treatment was characterized using an Olympus laser confocal microscope (OLS5000 model, Beijing, China). The microstructure of MAO (micro-arc oxidation) and EBR-MAO coatings at different pulse frequencies was observed using Hitachi ultra-high resolution scanning electron microscope (FE-SEM SUS8010, Guangzhou, China) and its accompanying Bruker D8 Advance energy dispersive spectrometer (EDS) (Shanghai, China). In addition, D8 Discoverer X-ray diffractometer (XRD) was used to analyze the phases of MAO and EBR-MAO coatings at different pulse frequencies.

The electrochemical performance test was conducted using the CorrTest CS350H electrochemical workstation (Wuhan, China), based on the classic three-electrode battery system: the sample was used as the working electrode (WE), the platinum plate was used as the auxiliary electrode (CE), and the saturated calomel electrode (RE) was used as the reference electrode. The electrochemical testing medium is a 3.5 wt.% NaCl solution. The testing includes alternating current impedance testing (EIS) and dynamic potential scanning testing. In AC impedance testing, the amplitude of the AC signal is 10 mV and the frequency range is 10^−1^~10^5^ Hz. In the dynamic potential scanning test, the potential scanning range is −3.5~0.5 V, and the scanning speed is 1 mV/s, in order to obtain the polarization curve and further analyze the corrosion resistance of the coating.

## 3. Results and Discussion

### 3.1. Cross-Sectional Morphology and Thickness of the Composite Coating

**Figure 2:**
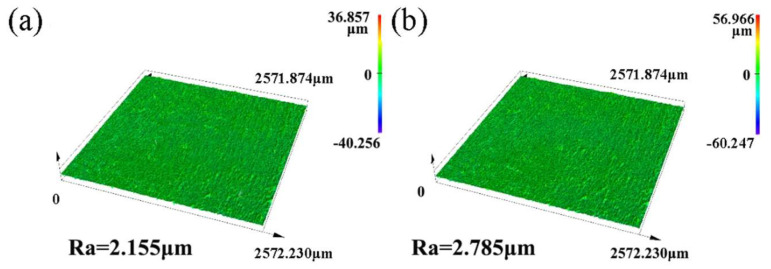
Three-dimensional surface morphology with and without remelting: (**a**) electron-beam remelting; (**b**) AZ31B.

Figure 2 shows the 3D morphology of the substrate surface with and without electron-beam remelting. It can be seen from the figure that there is not much difference in the 3D contour between the two, but the roughness of the substrate has decreased from 2.785 μm to 2.155 μm, indicating that the surface roughness of the substrate has been reduced after electron-beam remelting.

**Figure 3:**
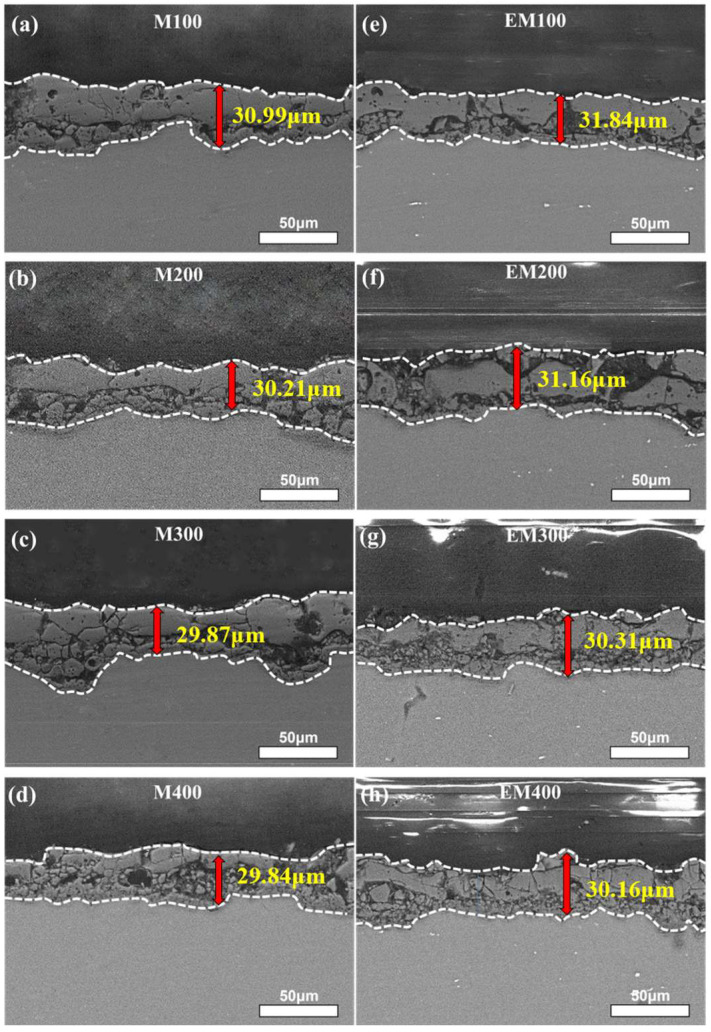
Cross-sectional morphologies of MAO coatings with and without beam remelting under different pulse frequencies: (**a**–**d**) M100, M200, M300, M400; (**e**–**h**) EM100, EM200, EM300, EM400.

Figure 3 presents the cross-sectional morphology of the MAO coatings and EBR-MAO coatings at different pulse frequencies. The bonding between coating and substrate is metallurgical bonding. The uneven interface observed in the MAO coating–substrate system can be attributed to the high temperature and pressure generated during the breakdown of the micro-arc oxidation film layer. This process causes localized melting of the film layer and substrate, resulting in an uneven metallurgical bond [27]. Notably, the interface between the EBR-MAO coating and the substrate is significantly smoother compared to that of the MAO coating and substrate. The EBR treatment refines the grains on the substrate surface and reduces the original surface roughness of the substrate. These modifications facilitate the formation of a denser layer, which subsequently influences the breakdown and discharge behavior during the MAO process, leading to a more uniform and compact coating structure.

**Figure 4:**
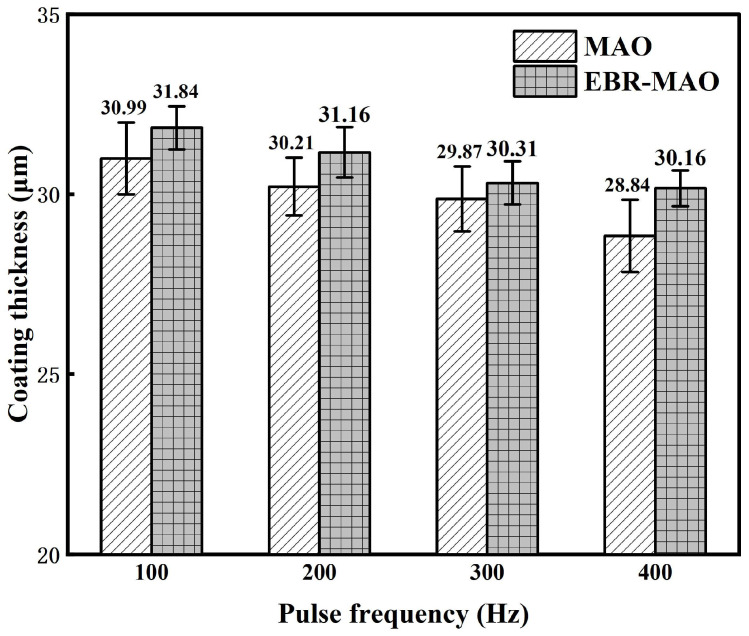
Thickness of MAO coating with and without electron-beam remelting under different pulse frequencies.

Figure 4 illustrates the average thickness of MAO coatings and EBR-MAO coatings at different pulse frequencies. The trend in thickness variation for both MAO and EBR-MAO coatings is consistent across the pulse frequencies. As the pulse frequency increases, the thickness of the coatings gradually decreases. This phenomenon can be attributed to the reduction in pulse duration within each cycle at higher frequencies, which leads to fewer discharges occurring at the same location and weaker discharge intensity. Weaker discharges result in lower temperatures and pressures within the discharge channels, reducing the amount of oxide that is melted, sputtered, and deposited. Consequently, at higher pulse frequencies, the growth rate of the coating is slower [15,21]. This trend highlights the critical role of pulse frequency in controlling the thickness and growth dynamics of MAO and EBR-MAO coatings.

### 3.2. Surface Morphology of the Composite Coating

**Figure 5:**
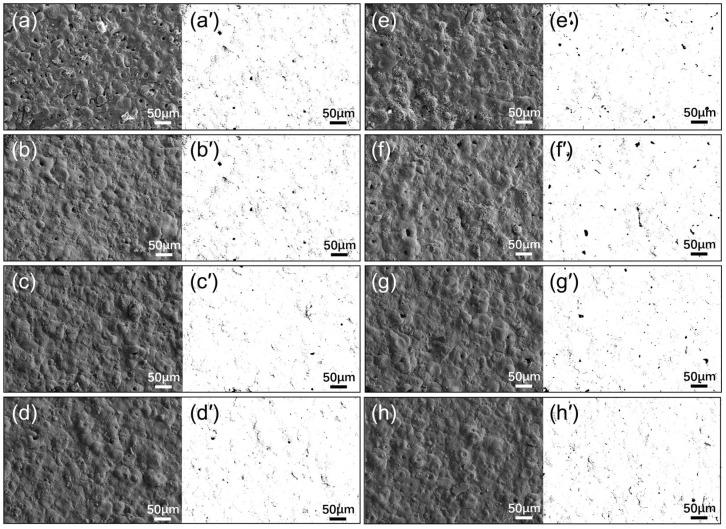
The surface morphology and pore distribution of MAO coatings with and without electron-beam remelting under different pulse frequencies: (**a**,**a′**–**d**,**d′**) M100, M200, M300, M400; (**e**,**e′**–**h**,**h′**) EM100, EM200, EM300, EM400.

Figure 5 presents the surface morphology of MAO coatings and EBR-MAO coatings at different pulse frequencies. The coated surface has numerous micropores, which are mostly circular or elliptical in shape. The microstructure of micro-arc oxidation (MAO) coatings, characterized by these micropores, is one of its distinctive features. The formation mechanism of these micropores is closely related to the localized high-temperature melting and rapid cooling processes during micro-arc discharge. The diameter of these micropores typically ranges from sub-micron to several microns, with the specific size largely dependent on process parameters (such as pulse frequency, voltage, electrolyte composition, etc.) and the substrate material. These micropores are usually unevenly distributed, varying in size and shape, and are primarily located on the surface and within the coating. At low pulse frequencies, the micropores are larger and unevenly distributed, potentially forming interconnected channels; whereas at high pulse frequencies, the size of the micropores decreases, their shape becomes more regular, distribution more uniform, the surface smoother, and they are predominantly sub-micron in size.

**Figure 6:**
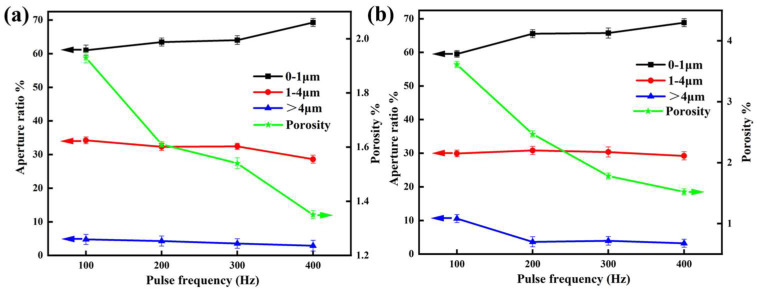
Surface porosity and pore size ratio of MAO coating with and without electron-beam remelting at different pulse frequencies: (**a**) EBR-MAO coatings; (**b**) MAO coatings.

Figure 6 illustrates the porosity and aperture ratio of these coatings, as analyzed using lmageJ 1.53c software. The results reveal that the porosity of EBR-MAO coatings is consistently lower than that of MAO coatings, with the porosity of EBR-MAO coatings reaching a minimum of 1.35% at 400 Hz. However, the porosity of both coatings varies with pulse frequency. At 100 Hz, the porosity of the EBR-MAO coating is significantly lower than that of the MAO coating. As the pulse frequency increases, the difference in porosity between the two types of coatings gradually diminishes. It can be attributed to the decrease in single-pulse duration and corresponding pulse energy at higher frequencies during the MAO process. As a result, the pores generated by each pulse become smaller, and the amount of molten material ejected from these pores decreases, leading to more uniform coating deposition.

In addition, the results show that with increasing pulse frequency, the proportion of micropores in the range of 0–1 μm gradually increases. When the pulse frequency increases from 100 to 300 Hz, the proportion of 0–1 μm pores approaches 60%, and at 400 Hz, it rises to nearly 70%. In contrast, the proportion of large pores (>4 μm) decreases significantly, while the proportion of medium-sized pores (1–4 μm) remains relatively stable. This indicates that high-frequency pulses help suppress the formation of large pores and promote the generation of finer micropores, resulting in a more uniform and compact coating structure. Meanwhile, the porosity shows a clear decreasing trend. As shown in Figure 6b, the porosity decreases from 3.61% to approximately 1.6%, and in Figure 6a, it decreases from 1.93% to about 1.3%. This suggests that increasing the pulse frequency can effectively reduce the overall porosity of the coating. This trend can be attributed to the lower energy and shorter duration of each discharge event at higher frequencies, which reduces intense local melting and surface ablation, thus favoring the formation of a stable, fine-pored ceramic layer.

Overall, appropriately increasing the pulse frequency is an effective strategy to optimize the pore structure of MAO coatings, enhancing both their compactness and corrosion resistance. Especially after electron-beam remelting, the coating’s microstructural uniformity and overall stability are further improved, providing a solid structural foundation for enhanced corrosion protection.

### 3.3. Composition of the Composite Coating

**Figure 7:**
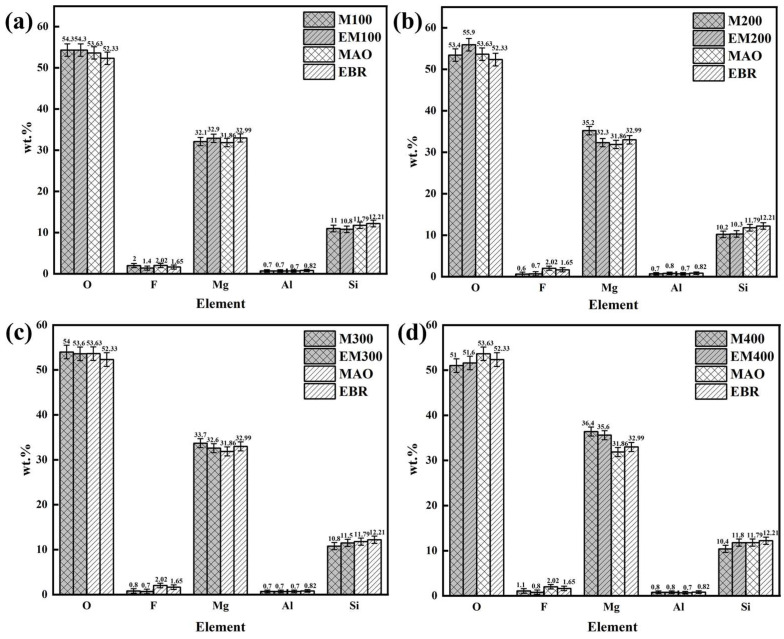
An analysis of the proportion of EDS elements in MAO coating with and without electron-beam remelting under different pulse frequencies. (**a**) 100 Hz, (**b**) 200 Hz, (**c**) 300 Hz, (**d**) 400 Hz.

Figure 7 shows the EDS elemental composition analysis of MAO and EBR-MAO coatings at pulse frequencies ranging from 100 Hz to 400 Hz. The results indicate that the relative content of Mg element in both MAO and EBR-MAO coatings reaches its maximum at 400 Hz. This indicates that when the pulse frequency is 400 Hz, more magnesium remains in the film during the micro-arc oxidation process rather than being ejected from the film surface.

Comparing the Si content in MAO and EBR-MAO coatings across different pulse frequencies, it is observed that the Si content does not change significantly after remelting at frequencies of 100–200 Hz. However, at 300–400 Hz, the increase in Si content becomes more pronounced. Specifically, at 400 Hz, the Si content in the EBR-MAO coating rises from 10.4% to 11.8% compared to the MAO coating. This increase in Si content indicates greater participation of the electrolyte in the reaction, which aligns with the observed increase in the thickness of the EBR-MAO coating compared to the MAO coating under the same parameters.

**Figure 8:**
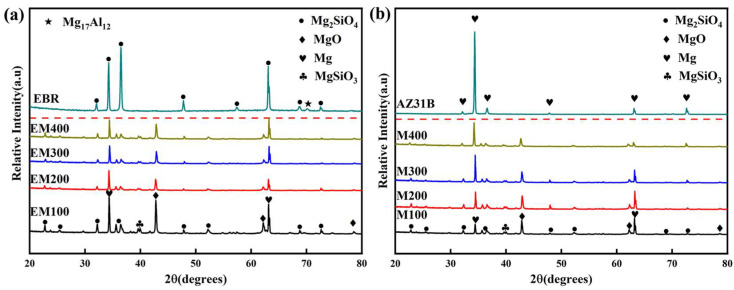
XRD patterns of MAO coating with and without electron-beam remelting under different pulse frequencies: (**a**) EBR-MAO coatings; (**b**) MAO coatings.

Additionally, Figure 8 includes the XRD patterns of MAO coatings at different pulse frequencies with and without electron-beam remelting. The analysis reveals that the phases present in the coatings remain consistent with and without remelting, primarily consisting of Mg, MgO, Mg_2_-SiO_4_, and Mg_2_-SiO_3_ phases. MgO is the first oxide formed during the micro-arc oxidation (MAO) process. It suppresses redox reactions and delays the penetration of corrosive media (such as Cl^−^) into the metallic substrate. Due to its extremely low electrical conductivity, MgO effectively increases the charge transfer resistance (R_t_) of the coating. However, MgO forms rapidly under MAO conditions and tends to have a porous structure, often accompanied by microcracks, which may serve as potential corrosion pathways. Mg_2_-SiO_4_ is typically formed through the reaction between SiO_3_^2−^ ions from the electrolyte and magnesium ions. It deposits in nanoscale or microscale dimensions within the microcracks and pores of the MgO layer, effectively sealing these defects and enhancing the overall coating density. As a high-hardness ceramic phase, Mg_2_-SiO_4_ possesses excellent structural stability, contributing to improved electrochemical stability. Furthermore, Mg_2_-SiO_4_ has very poor conductivity for both electrons and ions, thus further enhancing the impedance of the coating.

In summary, MgO plays a role in rapid film formation and provides basic protection, while Mg_2_-SiO_4_ contributes to densification and long-term stability. The synergistic effect of these two phases results in a high-performance MAO coating.

This consistency suggests that the porous structure of the MAO coatings allows X-rays to penetrate easily, corroborating the SEM analysis results. Furthermore, the pulse frequency does not significantly influence the phase composition of the EBR-MAO coatings. The Mg peaks observed in the XRD patterns are primarily attributed to the substrate.

### 3.4. Corrosion Resistance of the Composite Coating

**Figure 9:**
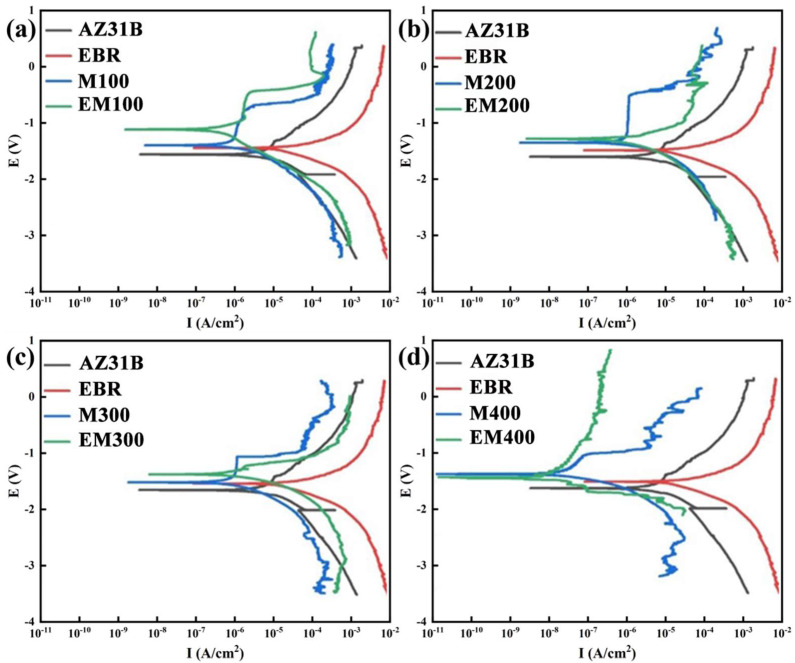
Polarization curves of MAO coating with and without electron-beam remelting under different pulse frequencies. (**a**) 100 Hz, (**b**) 200 Hz, (**c**) 300 Hz, (**d**) 400 Hz.

Figure 9 displays the potentiodynamic polarization curves of MAO and EBR-MAO coatings at different pulse frequency parameters, while Table 1 provides the fitted values for the self-corrosion potential E_corr_ and corrosion current density I_corr_. From Table 1, it is evident that the E_corr_ of the magnesium alloy substrate increased from −1.672 V to −1.521 V after electron-beam remelting. At pulse frequencies ranging from 100 Hz to 300 Hz, the E_corr_ of the EBR-MAO coating is higher than that of the MAO coating. Notably, at 100 Hz, the E_corr_ of the EBR-MAO coating reaches its highest value of −1.162 V.

Furthermore, at all tested pulse frequencies, the corrosion current density I_corr_ of the EBR-MAO coating is reduced by an order of magnitude compared to that of the MAO coating. At 400 Hz, the I_corr_ of the EBR-MAO coating reaches its lowest value of 2.897 × 10^−8^ A·cm^−2^. As shown in Figure 6a, the porosity of the coating also reaches its minimum value at 400 Hz. Combining the results from Figure 3h, it is clear that at 400 Hz, the coating exhibits the most complete bonding with the substrate, and the micro-discharge channels within the structure are uniformly distributed. These factors collectively contribute to the superior corrosion resistance of the EBR-MAO coating at this frequency.

**Figure 10:**
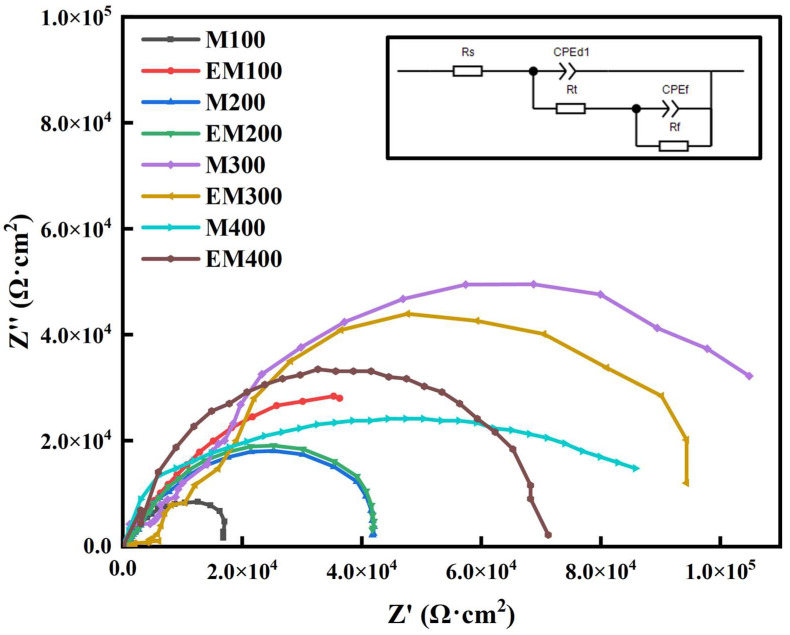
Nyquist curve of MAO coating with and without electron-beam remelting under different pulse frequencies.

Figure 10 presents the Nyquist curves of MAO and EBR-MAO coatings at different pulse frequencies. As observed, all samples exhibit typical semicircular characteristics, indicating that the charge transfer process plays a dominant role in the corrosion mechanism. In comparison, the samples treated with electron-beam remelting generally show larger semicircular radii, suggesting higher charge-transfer resistance (R_t_) and thus better corrosion resistance. Among them, the EM400 sample exhibits the largest impedance, indicating the best corrosion resistance, which may be attributed to its dense and uniform surface film that effectively hinders Cl^−^ ion penetration and localized corrosion.

To further quantitatively analyze the electrochemical behavior of the different samples, the equivalent circuit model shown in Figure 9 was used for fitting. The model consists of the following elements: R_s_ represents the solution resistance, R_t_ is the charge-transfer resistance, R_f_ denotes the film resistance, CPE_dl_ represents the constant phase element of the double layer, and CPE_f_ corresponds to the constant phase element of the surface film. The fitting results are presented in Table 2.

At pulse frequencies of 100 Hz and 400 Hz, the R_f_ value of EBR-MAO coatings increases by an order of magnitude compared to MAO coatings, indicating that the remelted layer significantly enhances long-term corrosion resistance. The R_f_ value reaches the highest value of 4.57 × 10^5^ Ω·cm^2^ at 400 Hz. In contrast, at pulse frequencies of 200 Hz and 300 Hz, the R_t_ and R_f_ values of the MAO coating with and without EBR show no significant difference, and the Nyquist curves reveal that the capacitance arc radii of the two coatings are similar.

Combining the insights from the polarization curves (Figure 9) and AC impedance spectra (Figure 10), it is clear that at a pulse frequency of 400 Hz, the MAO coating exhibits the lowest porosity, with most pores being micropores (0–1 μm), and demonstrates the best corrosion resistance. As is well known, once the breakdown potential is reached, micro-discharges occur at the metal/electrolyte interface. The presence of interconnected pores, particularly in coatings formed at lower pulse frequencies, can serve as channels for corrosive solutions to penetrate the coating. However, as the pulse frequency increases, the bonding at the metal/electrolyte interface becomes denser, and the coating’s compact structure effectively hinders the penetration of corrosive solutions, thereby significantly enhancing the anti-corrosion capability of the coating.

Pulse frequency has a significant influence on the microstructure and corrosion resistance of EBR-MAO composite coatings on magnesium alloys. In the composite coating system, the electron-beam remelting (EBR) process markedly improves the density of the substrate surface and refines the grain structure, providing a more stable interface foundation for the subsequent MAO treatment. As the MAO pulse frequency increases, the discharge behavior becomes more uniform and the discharge intensity weakens, resulting in fewer surface micropores, smaller pore sizes, and a denser outer-layer structure. These structural characteristics help inhibit the penetration and migration of corrosive media, particularly Cl^−^ ions, thereby slowing down the corrosion process.

**Figure 11:**
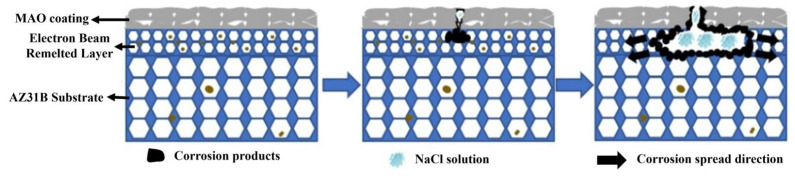
Corrosion mechanism diagram of EBR-MAO treated magnesium alloy.

In contrast, low-frequency discharge tends to cause localized overheating and the formation of larger pores, creating corrosion-sensitive regions that facilitate the penetration of Cl^−^ ions and the initiation of pitting corrosion at the coating–substrate interface. Under appropriate high-frequency conditions, the content of Mg, MgO, Mg_2_-SiO_4_, and Mg_2_-SiO_3_ phases in the coating increases, enhancing the bonding strength and forming a multilayered, multi-impedance protective system. This effectively improves the corrosion resistance of the composite coating. The corresponding corrosion mechanism is illustrated in Figure 11.

## 4. Conclusions

(1) Pulse frequency significantly influences the morphology of MAO and EBR-MAO coatings. As the pulse frequency increases, the proportion of micropores (0–1 μm) on the surface of EBR-MAO coatings rises from 61.03% to 69.27%, while the porosity gradually decreases from 1.93% to 1.35%. EBR-MAO coatings are denser especially at high frequency 400 Hz.
(2) The thickness of both MAO and EBR-MAO coatings decreases with increasing pulse frequency. Compared to MAO coatings, the thickness of the EBR-MAO coatings decreases slowly.
(3) The corrosion resistance of EBR-MAO coatings is consistently higher than that of MAO coatings at different pulse frequencies. At 400 Hz, I_corr_ of the EBR-MAO coating reaches its lowest value of 2.897 × 10^−8^ A·cm^−2^, and R_2_ reaches the highest value 4.57 × 10^5^ Ω·cm^2^.

These findings highlight the critical role of pulse frequency and electron-beam remelting in optimizing the microstructure, adhesion, and corrosion resistance of MAO coatings in magnesium alloys. This study investigated the effect of pulse frequency on the microstructure and corrosion resistance of electron-beam remelting–micro-arc oxidation (EBR-MAO) composite coatings on AZ31B magnesium alloy by controlling a single variable. In future work, orthogonal experiments can be conducted to explore the influence of multiple process parameters on the growth of MAO coatings. Additionally, pore-sealing treatment can be applied to the EBR-MAO coatings to further enhance their corrosion resistance.

## Figures and Tables

**Table 1:** Polarization-curve parameters of MAO coating with and without electron-beam remelting under different pulse frequencies.

	E_corr_/V	I_corr_/(A·cm^−2^)
AZ31B	−1.672	1.607 × 10^−5^
EBR	−1.521	1.144 × 10^−5^
M100	−1.460	2.012 × 10^−6^
EM100	−1.162	6.423 × 10^−7^
M200	−1.367	1.929 × 10^−6^
EM200	−1.295	5.681 × 10^−7^
M300	−1.487	1.802 × 10^−6^
EM300	−1.337	4.238 × 10^−7^
M400	−1.364	1.261 × 10^−7^
EM400	−1.438	2.897 × 10^−8^

**Table 2:** Nyquist fitting data of MAO coating with and without electron-beam remelting under different pulse frequencies.

	R_s_ (Ω·cm^2^)	CPE_d1_ (Ω^−1^s^n^cm^−2^)	N_1_	R_t_ (Ω·cm^2^)	CPE_f_ (Ω^−1^s^n^cm^−2^)	N_2_	R_f_ (Ω·cm^2^)
AZ31B	33.15	1.33 × 10^−6^	0.93	4.20 × 10^2^	1.25 × 10^−6^	0.91	1.64 × 10^3^
EBR	39.56	8.77 × 10^−6^	0.95	6.40 × 10^2^	4.99 × 10^−7^	1.06	1.69 × 10^3^
M100	48.30	1.38 × 10^−7^	0.72	4.60 × 10^4^	4.44 × 10^−8^	0.84	2.22 × 10^4^
EM100	35.26	3.24 × 10^−7^	0.78	6.08 × 10^4^	5.99 × 10^−7^	0.69	1.14 × 10^4^
M200	34.6	3.69 × 10^−8^	0.80	4.24 × 10^4^	2.22 × 10^−7^	0.79	3.05 × 10^4^
EM200	30.6	5.67 × 10^−8^	0.78	5.14 × 10^4^	2.80 × 10^−8^	0.86	4.81 × 10^4^
M300	44.6	4.62 × 10^−8^	0.84	7.54 × 10^4^	1.59 × 10^−8^	0.87	2.35 × 10^4^
EM300	34.2	3.54 × 10^−8^	0.80	8.34 × 10^4^	1.09 × 10^−8^	0.78	8.41 × 10^4^
M400	33.2	1.18 × 10^−7^	0.95	5.36 × 10^4^	3.97 × 10^−7^	0.89	4.15 × 10^4^
EM400	32.7	3.34 × 10^−7^	0.87	5.89 × 10^4^	3.97 × 10^−8^	0.79	4.57 × 10^5^

## Data Availability

The data that support the findings of this study are available from the corresponding author upon reasonable request.
